# Outcome comparison of patients who develop leptomeningeal disease or distant brain recurrence after brain metastases resection cavity radiosurgery

**DOI:** 10.1093/noajnl/vdab036

**Published:** 2021-03-02

**Authors:** Achiraya Teyateeti, Paul D Brown, Anita Mahajan, Nadia N Laack, Bruce E Pollock

**Affiliations:** 1 Department of Radiation Oncology, Mayo Clinic College of Medicine and Science, Rochester, Minnesota, USA; 2 Division of Radiation Oncology, Department of Radiology, Faculty of Medicine Siriraj Hospital, Mahidol University, Bangkok, Thailand; 3 Department of Neurologic Surgery, Mayo Clinic College of Medicine and Science, Rochester, Minnesota, USA

**Keywords:** brain metastases, cavity, leptomeningeal disease, radiation, stereotactic radiosurgery

## Abstract

**Background:**

To compare the outcomes between patients with leptomeningeal disease (LMD) and distant brain recurrence (DBR) after stereotactic radiosurgery (SRS) brain metastases (BM) resection cavity.

**Methods:**

Twenty-nine patients having single-fraction SRS after BM resection who developed either LMD (*n* = 11) or DBR (*n* = 18) as their initial and only site of intracranial progression were retrospectively reviewed.

**Results:**

Patients developing LMD more commonly had a metachronous presentation (91% vs 50%, *P* = .04) and recursive partitioning class 1 status (45% vs 6%, *P* = .02). There was no difference in the median time from SRS to the development of LMD or DBR (5.0 vs 3.8 months, *P* = .68). The majority of patients with LMD (10/11, 91%) developed the nodular variant (nLMD). Treatment for LMD was repeat SRS (*n* = 4), whole-brain radiation therapy (WBRT; *n* = 5), resection + WBRT (*n* = 1), and no treatment (*n* = 1). Treatment for DBR was repeat SRS (*n* = 9), WBRT (*n* = 3), resection + resection cavity SRS (*n* = 1), and no treatment (*n* = 5). Median overall survival (OS) from time of resection cavity SRS was 15.7 months in the LMD group and 12.7 months in the DBR group (*P* = .60), respectively. Median OS in salvage SRS and salvage WBRT were 25.4 and 5.0 months in the nLMD group (*P* = .004) while 18.7 and 16.2 months in the DBR group (*P* = .30), respectively.

**Conclusions:**

Following BM resection cavity SRS, nLMD recurrence is much more frequent than classical LMD. Salvage SRS may be considered for selected patients with nLMD, reserving salvage WBRT for patients with extensive intracranial disease without compromising survival. Further study with larger numbers of patients is needed.

**Key Points**

- Nodular leptomeningeal recurrence is common following postoperative stereotactic radiosurgery.
- Stereotactic radiosurgery is an effective salvage treatment in nodular leptomeningeal recurrence.

**Importance of the Study**

This study is one of the few which has compared the outcomes of brain metastases patients who develop LMD or DBR as the initial and only site of intracranial progression after postoperative SRS to resection cavity. We found nLMD was more common than classical LMD. For the entire cohort, we observed similar overall survival between patients with LMD and patients with DBR. Further analysis found that overall survival seemed to be longer in patients with nLMD receiving SRS compared to WBRT. These findings support that SRS is potentially an effective salvage treatment. A clinician may consider salvage SRS for selected patients with nLMD and reserve salvage WBRT for patients with extensive intracranial disease.

Stereotactic radiosurgery (SRS) or hypofractionated stereotactic radiotherapy (HSRT) following resection of brain metastases (BM) is commonly performed to reduce the chance of local tumor recurrence while maintaining cognitive function.^1–14^ Although postoperative radiation treatments vary widely with regard to timing, dose prescription, margin expansion, and number of fractions, local control (LC) rates typically range from 70% to 90% with radiation necrosis (RN) occurring in 5–10% of cases. Despite the improvement in LC compared to observation, patients remain at risk for leptomeningeal disease (LMD) and distant brain recurrence (DBR).

A recent meta-analysis that included 50 studies and 3458 patients reported that the chance of LMD was 13% following postoperative SRS/HSRT, and the incidence of DBR at 1 year was 47%.^15^ Whereas DBR has been accepted as a consequence of using SRS without whole-brain radiation therapy (WBRT) for many years,^16,17^ the observation of LMD following postoperative SRS/HSRT is relatively recent and is thought to be the result of tumor dissemination at the time of surgery. Numerous factors have correlated with an increased rate of LMD after postoperative SRS/HSRT including histology, tumor location, tumor morphology (solid vs cystic/hemorrhagic), presence of residual tumor, and method of tumor removal (en bloc vs piecemeal). Preoperative SRS has been utilized as a potential method to reduce the incidence of LMD after BM resection,^18–20^ and prospective trials are currently underway to better define the outcomes of patients having preoperative SRS (NCT01252797, NCT03398694, NCT02514915, NCT03750227).

It has been recognized that LMD noted after BM resection is distinct from the diffuse leptomeningeal carcinomatosis noted in approximately 4–15% of cancer patients.^21–23^ LMD after resection is generally focal nodules adherent to the dura or pia (nLMD), in contrast to classical LMD (cLMD) characterized by linear enhancement along brain surfaces or cranial nerves. In this study, we compared the presentation, treatment, overall survival (OS), and cause of death for BM patients developing either LMD or DBR as their initial and only site of intracranial progression after postoperative SRS.

## Materials and Methods

### Patients

The present study is a retrospective review approved by our institutional review board. All patients provided written consent for use of their data for scientific purposes. Between January 2010 and December 2019, 108 consecutive BM patients having surgical resection followed by postoperative SRS by the senior author (B.E.P.) were identified from a prospectively maintained registry. The pattern of intracranial progressions was assessed on a consensus basis by a radiation oncologist and neurosurgeon. Patients with either LMD or DBR as their initial and only site of intracranial progression were included. Patients were excluded if they refused research authorization (*n* = 5), had inadequate MRI follow-up (*n* = 9), had no intracranial progression after SRS (*n* = 49), had local recurrence (LR) alone (*n* = 7), LR with concurrent LMD or DBR (*n* = 1 and *n* = 4, respectively), or had concurrent LMD and DBR (*n* = 4). Twenty-nine patients with either LMD (*n* = 11) or DBR (*n* = 18) comprised the study population. The recursive partitioning analysis (RPA) classification^24^ and graded prognostic assessment (GPA) score^25^ were calculated for each patient.

### Treatment and Follow-up

Surgical resection was performed at the discretion of the neurosurgeon. SRS was performed in a single fraction in all cases using the Leksell Gamma Knife Perfexion (Elekta Instruments). Patients were immobilized by stereotactic headframe, followed by T1-weighted gadolinium-enhanced (T1Gd) and T2-weighted (T2W) MRI on a 1.5 T machine. Surgical cavities were outlined on the T2W MRI and any residual tumor or other BM identified with T1Gd MRI. A 2–3 mm margin was added to the resection cavity to obtain the clinical target volume (CTV).

Patients were recommended to have a clinical examination and MRI every 3 months for the first year after SRS unless clinically indicated at an earlier time, then every 3–6 months thereafter.

### Statistical Analysis

Follow-up MRI was reviewed for LR, LMD, DBR, and RN. LR was defined as a recurrent tumor within or contiguous with the CTV. LMD was either cLMD (defined as a new linear enhancement of the leptomeninges along cerebral sulci, cerebellar folia, cranial nerves, brainstem, or ependymal) or nLMD (defined as a new extra-axial nodular enhancement away from the resection cavity).^22,23^ DBR was defined as the development of new parenchymal tumors not present at the time of SRS. In order to minimize inconsistency in defining failure patterns, our study classified the patterns of intracranial progression based on the consensus of the radiation oncologist and neurosurgeon. The decision to proceed with additional treatment and the type of treatment was based on numerous factors including the nature of intracranial progression, the patients’ systemic disease status, the patients’ performance status, and patient preference. OS was calculated from the time of resection cavity SRS to death of any causes or last follow-up. The cause of death was classified as neurologic death (defined as death related to worsening of neurologic symptoms from intracranial progression and/or treatment of BM) or systemic death (defined as death related to worsening of symptoms and/or organ failures from extracranial progression and/or systemic treatment—defined as any chemotherapy, targeted therapy, hormonal therapy, or immunotherapy).

Descriptive statistics were reported as median/range for continuous variables and frequency/percent for categorical data. Non-parametric continuous variables between groups were compared with the Mann–Whitney *U*-test; categorical variables were compared by the Fisher’s exact test. Time-to-event analyses were estimated by the Kaplan–Meier method. Log-rank testing was performed to compare OS between groups. Statistical analysis was performed by SPSS version 21 (IBM Corp.).

## Results

### Characteristics at the Time of Initial Diagnosis of BM and Postoperative SRS

The characteristics of the 29 patients at the time of initial diagnosis of BM and postoperative SRS are outlined in Table 1. A comparison of characteristics between patients with LMD and DBR was given in Table 2. Patients developing LMD more commonly had a metachronous presentation (91% vs 50%, *P* = .04) and RPA class 1 status (45% vs 6%, *P* = .02), but the groups were similar with regard to systemic disease, tumor location, and tumor size. Patients with LMD more often had a single BM (73% vs 33%) and breast and non-small cell lung cancer histology, but these did not reach statistical significance (*P* = .06 and *P* = .47). Regarding the treatment at the time of initial diagnosis of BM, the median time from tumor resection to SRS was 6 days (range, 2–49). At the time of postoperative SRS, 5 patients (17%) had residual tumor at resection cavity. A median of 7 isocenters (range, 1–16) were used to cover a median CTV of 7.4 cm^3^ (range, 1.4–29.5). The median prescribed dose to the CTV was 18 Gy (range, 12–18); the median maximum dose was 36 Gy (range, 22.5–40.0). Fifteen patients (52%) had additional BM treated (median, 2 tumors). The tumor margin dose for the additional tumors ranged from 18 to 22 Gy. No difference was noted in the CTV, margin dose, or maximum dose between the groups (Table 2). Systemic treatments given at the time of initial diagnosis of BM and/or prior to intracranial progression after resection cavity SRS were more common among patients with DBR (39% vs 9%) without statistical significance (*P* = .11).

**Table 1.** Characteristics at the Time of Initial Diagnosis of Brain Metastases and Postoperative Stereotactic Radiosurgery

ID	Age (years)	Sex	Primary Tumor	Systemic Disease	Timing of BM (Time After Primary Diagnosis)	Number of BM	RPA	GPA	Tumor Location	Resected Tumor Size (cm)	Resected Tumor Volume (cm^3^)	CTV (cm^3^)	RT Dose (Gy)	Max RT Dose (Gy)
*Leptomeningeal disease*														
1	84	Male	NSCLC	Uncontrolled	Synchronous	1	2	1.5	Cerebellar	3.8	12.8	8.90	17.0	34.0
2	62	Female	NSCLC	Controlled	Metachronous (9 months)	1	1	2.5	Cerebral	4.7	48.0	6.90	18.0	36.0
3	75	Female	Breast	Controlled	Metachronous (185 months)	1	2	2.0	Cerebral	4.5	30.7	8.50	18.0	36.0
4	81	Female	NSCLC	Controlled	Metachronous (10 months)	1	3	2.0	Cerebellar	2.9	8.8	4.50	18.0	36.0
5	74	Female	NSCLC	Controlled	Metachronous (35 months)	1	2	3.0	Cerebral	2.5	12.1	6.90	18.0	36.0
6	49	Female	Breast	Controlled	Metachronous (25 months)	1	1	4.0	Cerebral	3.9	24.5	7.40	18.0	36.0
7	44	Female	Breast	Controlled	Metachronous (22 months)	1	1	4.0	Cerebellar	2.8	11.4	11.50	15.0	37.5
8	58	Male	Colon	Controlled	Metachronous (76 months)	3	1	4.0	Cerebral	3.4	8.6	9.90	15.0	30.0
9	82	Male	NSCLC	Controlled	Metachronous (23 months)	3	2	2.5	Cerebral	3.3	9.6	4.80	18.0	40.0
10	43	Male	NSCLC	Uncontrolled	Metachronous (9 months)	2	2	2.5	Cerebral	4.1	31.2	12.70	15.0	30.0
11	65	Female	Ovary	Uncontrolled	Metachronous (14 months)	1	2	2.0	Cerebral	1.9	3.6	6.2	18.0	36.0
*Distant brain recurrence*														
12	65	Female	Breast	Controlled	Metachronous (48 months)	1	2	3.0	Cerebral	2.1	3.5	2.10	18.0	22.5
13	71	Female	NSCLC	Uncontrolled	Synchronous	1	2	2.0	Cerebral	5.3	37.6	9.00	16.0	32.0
14	57	Male	NSCLC	Controlled	Metachronous (7 months)	2	1	3.0	Cerebral	1.4	1.4	2.30	18.0	25.7
15	59	Female	NSCLC	Uncontrolled	Synchronous	3	2	3.0	Cerebral	2.1	5.4	3.90	18.0	36.0
16	72	Female	NSCLC	Uncontrolled	Synchronous	2	2	1.5	Cerebral	3.8	18.6	8.00	18.0	36.0
17	68	Female	SCLC	Uncontrolled	Synchronous	1	2	3.0	Cerebellar	4.7	25.5	13.50	15.0	30.0
18	65	Female	RCC	Uncontrolled	Synchronous	2	3	0.5	Cerebral	2.0	3.5	4.40	18.0	36.0
19	52	Male	Melanoma	Controlled	Metachronous (16 months)	3	2	1.5	Cerebral	3.5	17.9	5.10	18.0	36.0
20	77	Female	Colon	Controlled	Metachronous (20 months)	2	2	2.0	Cerebral	3.8	14.3	12.40	15.0	30.0
21	76	Female	Breast	Controlled	Metachronous (246 months)	1	2	2.5	Cerebral	1.4	1.6	1.40	18.0	36.0
22	57	Male	RCC	Uncontrolled	Synchronous	2	2	1.5	Cerebral	3.3	7.7	14.20	15.0	30.0
23	39	Female	Melanoma	Uncontrolled	Metachronous (44 months)	3	2	2.0	Cerebral	3.7	21.4	10.60	16.0	32.0
24	64	Female	NSCLC	Uncontrolled	Synchronous	2	2	1.0	Cerebellar	3.9	30.2	12.00	16.0	32.0
25	61	Female	NSCLC	Uncontrolled	Synchronous	11	2	0.5	Cerebellar	3.1	13.8	5.40	18.0	36.0
26	32	Female	Rectum	Uncontrolled	Metachronous (40 months)	3	2	2.5	Cerebellar	3.3	10.3	6.20	18.0	36.0
27	62	Male	NSCLC	Uncontrolled	Synchronous	1	2	1.5	Cerebral	3.0	13.7	29.50	12.0	26.7
28	36	Male	Melanoma	Controlled	Metachronous (90 months)	1	2	2.5	Cerebral	4.5	62.6	4.90	18.0	36.0
29	69	Female	Breast	Controlled	Metachronous (188 months)	6	2	1.0	Cerebral	4.3	28.0	8.80	18.0	36.0

NSCLC, non-small cell lung cancer; BM, brain metastases; RPA, recursive partitioning analysis; GPA, graded prognostic assessment; CTV, clinical target volume; RT, radiation therapy.

**Table 2.** Comparison of Characteristics at the Time of Initial Diagnosis and Postoperative Stereotactic Radiosurgery

Factor	LMD (*n* = 11)	DBR (*n* = 18)	*P* value
Median age, years (range)	65 (43–84)	63 (32–77)	.36
Sex			
Male	4 (36%)	5 (28%)	.69
Female	7 (64%)	13 (72%)	
Primary tumor			
NSCLC	6 (55%)	7 (39%)	.47
Melanoma	0 (0%)	3 (17%)	
Breast	3 (27%)	2 (11%)	
Renal cell carcinoma	0 (0%)	2 (11%)	
Other	2 (18%)	4 (22%)	
Systemic disease			
Controlled	8 (73%)	7 (39%)	.69
Uncontrolled	3 (27%)	11 (61%)	
BM presentation			
Synchronous	1 (9%)	9 (50%)	.04
Metachronous	10 (91%)	9 (50%)	
BM number			
Single	8 (73%)	6 (33%)	.06
Multiple	3 (27%)	12 (67%)	
RPA			
1	5 (45%)	1 (6%)	.02
2	5 (45%)	16 (88%)	
3	1 (9%)	1 (6%)	
GPA			
0.5–2.0	4 (36%)	11 (61%)	.26
2.5–4.0	7 (64%)	7 (39%)	
Location of resected tumor			
Cerebral hemisphere	8 (73%)	14 (78%)	1.00
Cerebellar	3 (27%)	4 (22%)	
Tumor features			
Solid	4 (36%)	7 (39%)	1.00
Cystic	3 (27%)	4 (22%)	
Mixed solid-cystic	3 (27%)	3 (17%)	
Hemorrhagic	1 (9%)	4 (22%)	
Pial involvement			
Yes	8 (73%)	14 (78%)	1.00
No	3 (27%)	4 (22%)	
Resected tumor size			
≤3 cm	4 (36%)	6 (33%)	1.00
>3 cm	7 (64%)	12 (67%)	
Median resected tumor volume, cm^3^ (range)	12.1 (3.6–48.0)	14.0 (1.4–62.6)	.90
Median number of isocenters (range)	7 (1–11)	7 (1–16)	.88
Median CTV, cm^3^ (range)	7.4 (4.5–12.7)	7.1 (1.4–29.5)	.81
Median margin dose, Gy (range)	18 (15–18)	18 (12–18)	.68
Median maximum dose, Gy (range)	36 (30–40)	34 (22.5–36)	.58

LMD, leptomeningeal disease; DBR, distant brain recurrence; NSCLC, non-small cell lung cancer; BM, brain metastases; GPA, graded prognostic assessment; RPA, recursive partitioning analysis; CTV, clinical target volume.

### Characteristics and Treatments at the Time of Intracranial Progression

Characteristics of 29 patients in detail were given in Table 3. There was no difference in the time from resection cavity SRS to the development of LMD (median 5.0 months; range, 2–10) or DBR (median 3.8 months; range, 2–21; *P* = .68). Uncontrolled systemic disease at the time of initial intracranial progression was more common among patients with DBR (44% vs 18%) without statistical significance (*P* = .23).

**Table 3.** Characteristics at the Time of Initial Intracranial Progression, Subsequent Treatment, and Treatment Outcomes

ID	Time to Intracranial Progression (months)	Systemic Disease at the Time of Intracranial Progression	Initial Treatment of Intracranial Progression	Summary of Treatment Following Intracranial Progression (in Sequence)	Overall Survival (months)	Survival After Intracranial Progression (months)	Status at Last Follow-up/Cause of Death
*Leptomeningeal disease*							
1	3	Controlled	SRS	SRS, SRS	27	24	Alive
2	2	Controlled	Surgery + WBRT	Surgery, WBRT	4	2	Alive
3	10	Uncontrolled	SRS	SRS, systemic	22	12	Alive
4	9	Controlled	—	—	12	3	Neurologic
5	3	Controlled	WBRT	WBRT, SRS,SRS,SRS	25	22	Neurologic
6	10	Controlled	WBRT	WBRT and spinal RT, systemic	14	3	Neurologic
7	5	Controlled	WBRT	WBRT, SRS, systemic	25	20	Neurologic
8	6	Controlled	WBRT	WBRT	10	4	Neurologic
9	3	Controlled	WBRT	WBRT	5	2	Neurologic
10	5	Uncontrolled	SRS	SRS, systemic	16	11	Systemic
11	3	Controlled	SRS	SRS	18	15	Alive
*Distant brain recurrence*							
12	8	Controlled	SRS	SRS, systemic	19	11	Alive
13	21	Uncontrolled	SRS	SRS, systemic	24	3	Alive
14	3	Controlled	WBRT	WBRT	12	9	Alive
15	5	Controlled	SRS	SRS, SRS, systemic	26	21	Alive
16	15	Controlled	—	—	15	0	Alive
17	3	Controlled	SRS	SRS, WBRT	19	16	Neurologic
18	4	Uncontrolled	WBRT	WBRT, systemic	16	12	Neurologic
19	2	Controlled	WBRT	WBRT, systemic	5	3	Neurologic
20	3	Controlled	SRS	SRS, WBRT	13	10	Neurologic
21	10	Controlled	Surgery + SRS	Surgery, SRS	11	1	Systemic
22	2	Uncontrolled	—	—	3	1	Systemic
23	12	Uncontrolled	Systemic	Systemic	12	0.5	Systemic
24	3	Controlled	SRS	SRS, systemic	10	7	Systemic
25	9	Uncontrolled	SRS	SRS, SRS, systemic	20	11	Systemic
26	3	Uncontrolled	Systemic	Systemic	5	2	Systemic
27	4	Uncontrolled	SRS	SRS, systemic	6	2	Systemic
28	4	Controlled	SRS	SRS, systemic	55	51	Systemic
29	4	Uncontrolled	—	—	8	4	Systemic

SRS, stereotactic radiosurgery; WBRT, whole-brain radiation therapy; RT, radiation therapy.

**Figure 1.**
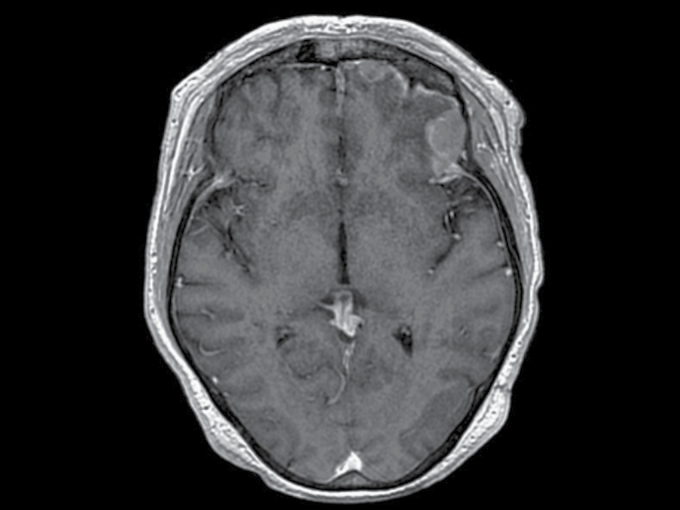
An MRI demonstrates nodular leptomeningeal recurrences (2 nodules) at the left frontal lobe adjacent to the surgical cavity in a 62-year-old non-small cell lung cancer patient receiving brain metastasis resection and cavity stereotactic radiosurgery 2 months ago.

Follow-up after resection cavity SRS found that 11 patients had nLMD (*n* = 10) or cLMD (*n* = 1, ID 6) diagnosed as their initial and only site of intracranial progression. Figure 1 demonstrates an MRI of a patient having nLMD adjacent to the surgical cavity. Ten patients had additional radiation for the LMD including WBRT (*n* = 5), SRS (*n* = 4), and resection + WBRT (*n* = 1). Three patients had further treatment for intracranial progression. One patient (ID 5) developed new nLMD after WBRT and underwent 3 additional SRS procedures at 11, 14, and 17 months after radiation therapy. Two patients (ID 1 and 7) underwent repeat SRS to treat new nLMD at 16 and 11 months, respectively. Remarkably, both initial and subsequent nLMD recurrences developed at or adjacent to the surgical cavity and/or previously irradiated site, irrespective of previously receiving WBRT or SRS.

Eighteen patients had DBR diagnosed as their initial and only site of intracranial progression after resection cavity SRS. Thirteen patients had additional radiation for DBR including WBRT (*n* = 3), SRS (*n* = 9), and resection + cavity SRS (*n* = 1). Four patients had further treatment for intracranial progression. Two patients developed new cLMD (ID 17) or nLMD (ID 20) at 2 and 8 months, respectively, and underwent WBRT. One patient (ID 15) underwent repeat SRS at 3 months for DBR, and one patient (ID 25) underwent 2 additional SRS procedures for DBR at 9 and 14 months.

After intracranial progression, patients with DBR were likely to receive systemic treatments compared to patients with LMD (44% vs 36%); however, there was no statistical significance (*P* = .72). Overall, systemic treatments given at any time were more common among patients with DBR (61% vs 36%) without statistical significance (*P* = .26) as well.

### Outcomes of Intracranial Progression Treatment

**Figure 2.**
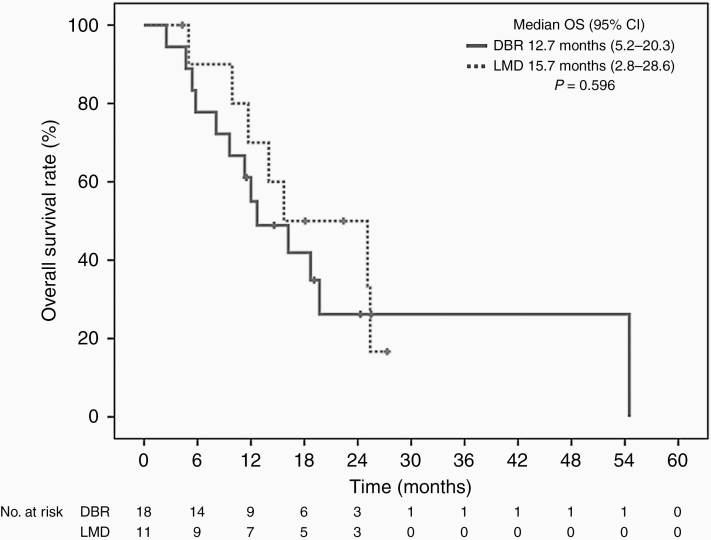
Overall survival in patients with different patterns of intracranial progression: leptomeningeal disease (classical variant and nodular variant) versus distant brain recurrence. LMD, leptomeningeal disease; DBR, distant brain recurrence; OS, overall survival; CI, confidence interval.

As given in Table 3, systemic death was significant higher among patients with DBR (50% vs 9%; *P* = .04) while neurologic death was more common among patients with LMD without statistical significant (55% vs 22%; *P* = .11). For the entire cohort, there was no difference in OS for patients with LMD (median 15.7 months) or DBR (median 12.7 months; *P* = .60; Figure 2). Median survival after initial intracranial progression was longer in LMD patients (15.4 vs 9.9 months), but there was no statistical significance (*P* = .58).

Owing to the difference in disease characteristics between LMD variants and possible influence on treatment, survival analysis of nLMD comparing to DBR was further performed. There was no statistically significant difference in OS between patients with nLMD (median 25.1 months) and patients with DBR (median 12.7 months; *P* = .52). Also, there was no significant difference in median survival after initial intracranial progression between patients with nLMD and patients with DBR (15.4 months vs 9.9 months; *P* = .45).

**Figure 3.**
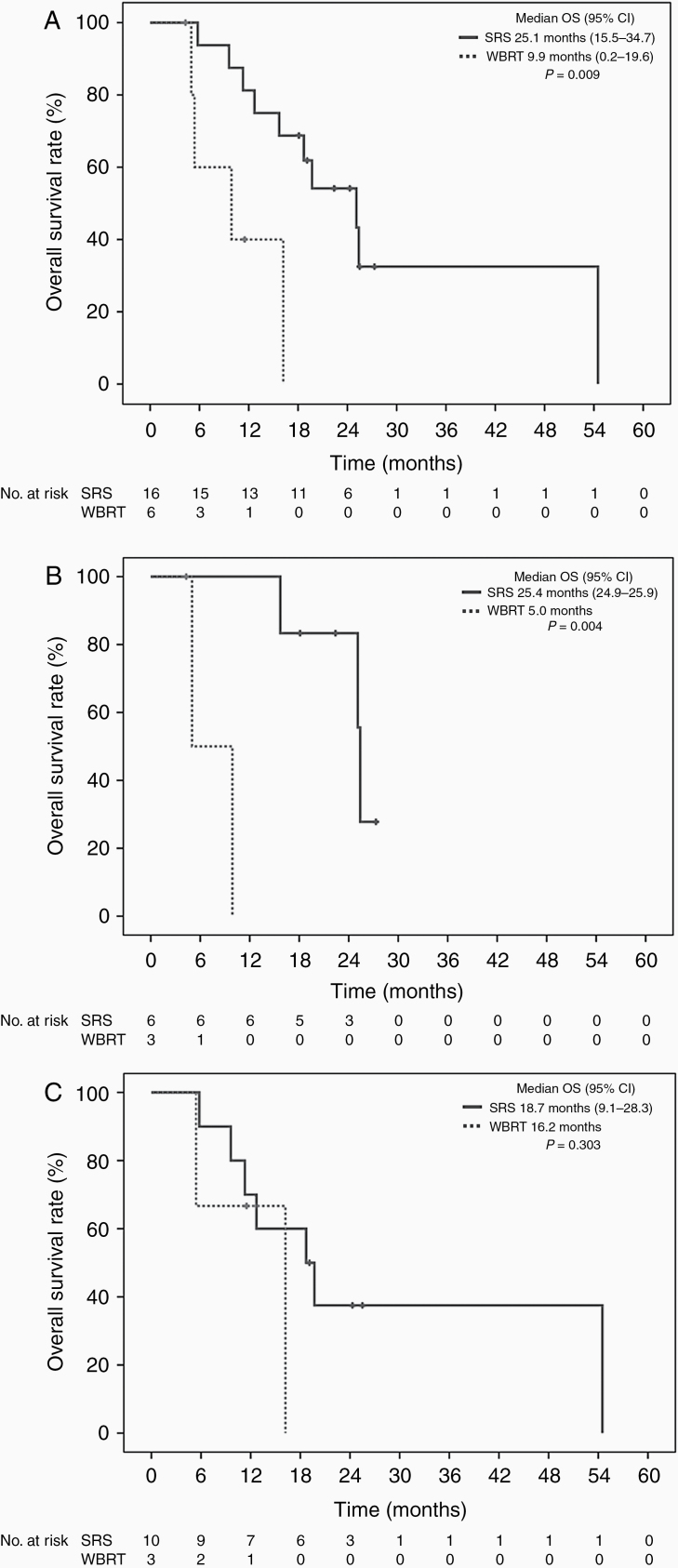
(A) Overall survival in patients with different salvage treatment: stereotactic radiosurgery versus whole-brain radiation therapy. (B) Overall survival in nodular leptomeningeal patients with different salvage treatment: stereotactic radiosurgery versus whole-brain radiation therapy. (C) Overall survival in distant brain recurrence patients with different salvage treatment: stereotactic radiosurgery versus whole-brain radiation therapy. SRS, stereotactic radiosurgery; WBRT, whole-brain radiation therapy; OS, overall survival; CI, confidence interval.

Regarding the effect of salvage radiation on survival, for the entire cohort, receiving salvage SRS at any time of intracranial progression was associated with significantly longer OS (median 25.1 months vs 9.9 months; *P* = .009; Figure 3A) and survival after initial intracranial progression (median 19.7 months vs 4.4 months; *P* = .046) compared to salvage WBRT. For nLMD patients, salvage SRS for either initial or subsequent nLMD related to significant longer OS (median 25.4 months vs 5.0 months; *P* = .004; Figure 3B) and survival after initial intracranial progression (median 22.0 months vs 4.4 months; *P* = .009) compared to WBRT with or without upfront surgery. However, there was no significant difference in OS (median 18.7 months vs 16.2 months; *P* = .30; Figure 3C) and survival after initial intracranial progression (median 10.9 months vs 12.4 months; *P* = .69) in patients receiving salvage SRS for DBR compared to WBRT alone.

## Discussion

Surgical resection of BM is often required for patients with large symptomatic lesions and has been shown to improve survival in patients with a solitary tumor.^26^ Historically, postoperative WBRT was given to improve LC, but prospective trials have documented cognitive deterioration for patients receiving WBRT in comparison to those having SRS alone.^2,27^ Over the past 15 years, postoperative SRS/HSRT after BM resection has emerged as an accepted option to improve LC while avoiding the cognitive decline associated with WBRT. However, the omission of WBRT after surgical resection has been related to an increase not only in DBR requiring further treatment, but also more frequent instances of LMD.

Cagney et al.^21^ compared 318 patients having resection and either postoperative SRS or SRT to 870 patients having radiation alone. They found that the patients having surgery were at increased risk for pachymeningeal seeding (defined similar to nLMD as nodular, enhancing tumors arising from the dura or pia), with an incidence of 8.4%, compared to 0% for patients having only radiation. No difference was noted in the chance of LMD (defined similar to cLMD as enhancement in the subarachnoid spaces overlying the brain or cranial nerves) between the groups.

Prabhu et al.^22^ performed a multi-institutional analysis of LMD after BM resection cavity SRS/HSRT including 147 patients from 7 centers. The median time from postoperative SRS/HSRT to LMD was 5.6 months. Eighty-four patients (57%) had nLMD and 63 patients (43%) had cLMD. Patients with cLMD more commonly were symptomatic compared to patients with nLMD (71% vs 51%), underwent WBRT more frequently as salvage therapy (40/42, 95% vs 35/73, 48%), and had shorter median OS (3.3 months vs 8.2 months). Multivariable analysis of 115 patients receiving salvage radiation treatment found that the pattern of LMD (nLMD vs cLMD) was a predictor of OS. Similarly, the current study found that nLMD recurrence after postoperative SRS was more common than cLMD (91% vs 9%). Additionally, OS in patients with nLMD (median 25.1 months) was longer than patients with cLMD (14.1 months).

Although the current study found no difference in OS for patients with either LMD or DBR as their initial and only site of intracranial progression after BM resection cavity SRS, this finding should be cautiously interpreted owing to the limited number of patients, heterogeneity of BM patients at the time of initial diagnosis and at the time of intracranial progression, lack of detailed progression-free survival outcomes after salvage therapy, and differences in nature of disease thereby inevitably had an effect on treatment selections. The patient groups differed with regard to the initial presentation (synchronous vs metachronous), but were otherwise similar in primary tumor type, tumor size, and location. Similar to Cagney et al.,^21^ patients with LMD more commonly had RPA class 1 disease, and in the absence of extracranial systemic disease, they more often died of neurologic causes compared to patients with DBR.

Owing to inherent differences in LMD types as previously mentioned, we further explored the nLMD group. We observed that 11 of 13 patients (85%) in the entire study who developed LMD had the nLMD variant and new nLMD likely to be developed at or adjacent to the surgical cavity and/or previously irradiated site. Consequently, we hypothesized that focal radiation like SRS is potentially adequate for LC, and as our experience with managing these patients grew, we transitioned from using WBRT to repeat SRS as salvage therapy whenever possible. Practice changing at our institute is in line with EANO–ESMO clinical practice guidelines recommending focal radiation for type B LMD (nodular LMD) and possibly be an option for type C LMD (mixed linear and nodular LMD).^28^ Recent study from Prabhu et al.^22^ also supported that repeat SRS is a sound treatment alternative to WBRT for patients with limited nLMD. Additionally, the current study demonstrated that survival after initial intracranial progression in patients with nLMD receiving SRS was longer than receiving WBRT, corresponding to the results from Prabhu et al.^22^ Based upon these findings, we suggest that SRS may be considered as a treatment option for selected patients with nLMD, while WBRT may be reserved for patients with cLMD, patients with extensive nLMD, or patients with a large number of DBR, whenever possible to maintain patients’ neurocognitive function.

This study is subject to the limitations of any retrospective study, especially selection bias in deciding the type of treatment for patients with intracranial disease progression. The number of patients is small so a detailed analysis of factors predisposing patients for either LMD or DBR, and determination of the optimal salvage therapy, was not possible. Additionally, with the recent great advancement in systemic therapy, OS in this study may not best reflect the outcomes of current practice. Modern targeted therapy and/or immunotherapy studies have shown impressive intracranial response and/or improvement in survival outcomes of patients with BM or LMD.^29–32^ Further study focusing on combining these agents with radiotherapy, particularly SRS, should be warranted.

## Conclusions

Though the transition from postoperative WBRT to cavity SRS after BM resection preserves neurocognitive function, it is associated with an increased risk of DBR and LMD, either cLMD or the more common nLMD variant. Patients with LMD or DBR could be effectively salvaged with either SRS or WBRT; however, survival seemed to be longer with salvage SRS compared to salvage WBRT in patients with nLMD. Suggesting that salvage SRS may be considered for selected patients such as limited nLMD and reserve salvage WBRT for patients with extensive intracranial disease, similar to the management of patients with DBR.

## Notes

**Authorship Statement** Conception and design: B.E.P. and P.D.B.; data acquisition and analysis: A.T. and B.E.P.; drafting manuscript: A.T. and B.E.P.; revising manuscript: A.T., P.D.B., A.M., N.N.L., and B.E.P.; final approval: A.T., P.D.B., A.M., N.N.L., and B.E.P.
